# Loss of pulmonary capillaries in idiopathic pulmonary arterial hypertension with low diffusion capacity is accompanied by early diffuse emphysema detected by ^129^Xe MRI

**DOI:** 10.1007/s00330-024-11209-1

**Published:** 2024-12-08

**Authors:** Agilo Luitger Kern, Da-Hee Park, Jan Fuge, Jens M. Hohlfeld, Frank Wacker, Marius M. Hoeper, Karen M. Olsson, Jens Vogel-Claussen

**Affiliations:** 1 Institute for Diagnostic and Interventional Radiology, Hannover Medical School, Hannover, Germany; 2 Biomedical Research in Endstage and Obstructive Lung Disease Hannover (BREATH), German Center for Lung Research (DZL), Hannover, Germany; 3 Department of Respiratory Medicine and Infectious Diseases, Hannover Medical School, Hannover, Germany; 4 Clinical Airway Research, Fraunhofer Institute for Toxicology and Experimental Medicine (ITEM), Hannover, Germany

**Keywords:** Pulmonary arterial hypertension, Pulmonary circulation, Pulmonary emphysema, Magnetic resonance imaging, Xenon

## Abstract

**Objectives:**

Recent studies suggest the existence of an idiopathic pulmonary arterial hypertension (IPAH) phenotype affecting mostly patients with a smoking history, characterised by low diffusion capacity for carbon monoxide (*D*_LCO_) without clinically significant emphysema. This study’s objective was to test the hypothesis of a loss of pulmonary capillaries as an underlying mechanism by comparison to other patient groups with and without pulmonary hypertension (PH).

**Materials and methods:**

Between March 2019 and June 2023, patients of four groups were recruited for this observational study: IPAH with preserved (1) and low *D*_LCO_ (2), combined pulmonary fibrosis and emphysema with PH (3), and emphysema without PH (4). Patients underwent clinical CT and ^129^Xe MRI including dissolved-phase imaging yielding the ratio of ^129^Xe in red blood cells and membrane tissues (RBC-M), chemical shift saturation recovery for determining RBC fraction η and diffusion-weighted imaging yielding surface-volume ratio. Kruskal–Wallis tests were used for statistical analysis.

**Results:**

Twenty-nine participants were recruited, of which 22 (age 64 ± 10, 11 male, 5/5/7/5 for the individual groups) could be included in the analysis. RBC-M and η were reduced in IPAH with low versus preserved *D*_LCO_ and emphysema groups (*p* ≤ 0.01). CT low-attenuation area percentage was not increased in IPAH with low *D*_LCO_ compared to any group. ^129^Xe MRI-derived surface-volume ratio was reduced in IPAH with low versus preserved *D*_LCO_ (*p* = 0.04).

**Conclusion:**

Results are consistent with a loss of pulmonary capillaries in patients with IPAH and low *D*_LCO_ along with destruction of alveolar tissue, likely due to early diffuse emphysema.

**Key Points:**

***Question*** *A loss of pulmonary capillaries has been suggested in patients with IPAH and low diffusion capacity without clinically significant emphysema on CT*.

***Findings*** ^*129*^*Xe uptake in red blood cells and lung surface-volume ratio were reduced in IPAH patients with low compared to preserved diffusion capacity*.

***Clinical relevance*** *This study furthers the understanding of the underlying pathological mechanisms in IPAH with low diffusion capacity, providing evidence that loss of pulmonary capillaries is accompanied by alveolar tissue destruction despite near-normal CT*.

## Introduction

Idiopathic pulmonary arterial hypertension (IPAH) is a rare disease of the pulmonary vasculature characterised by pre-capillary pulmonary hypertension (PH) and can only be classified in the absence of other causes for PH [1]. Historically, IPAH predominantly affected young women. However, potentially due to changed demographics, IPAH is nowadays frequently diagnosed in elderly patients with cardiopulmonary comorbidities [2]. Among these, a distinct “lung phenotype” has emerged consisting of predominantly male patients with low diffusion capacity of the lung for carbon monoxide (*D*_LCO_) and significant smoking history. Despite frequent hypoxaemia, lung function testing and CT show no or minimal signs of parenchymal lung disease [3, 4]. This phenotype is associated with high mortality and distinct from other diseases associated with pulmonary arterial hypertension and low *D*_LCO_ like pulmonary veno-occlusive disease, pulmonary capillary haemangiomatosis or systemic sclerosis.

Whereas smoking tobacco is not considered a risk factor for pulmonary arterial hypertension, long-standing tobacco exposure is hypothesised to cause direct damage to the alveolar-capillary membrane. Mice exposed to tobacco smoke showed endothelial cell apoptosis and loss of pulmonary capillaries [5]. Histopathology from 24 IPAH patients with a lung phenotype showed vascular pruning with capillary rarefication [6]. Direct damage to the alveolar-capillary membrane resulted in the loss of small pulmonary vessels and the term “vanishing pulmonary capillary syndrome” was proposed for this condition [7].

Smoking effects on the lungs were described in other diagnoses like chronic obstructive pulmonary disease (COPD) and combined pulmonary fibrosis and emphysema (CPFE) [8]. Recently, a vascular phenotype of COPD has been described [1]. There is typically only mild pulmonary arterial pressure elevation in COPD. Yet, a COPD subgroup with poor survival exists, characterised by moderate airflow limitation, severe precapillary PH, relatively low PaCO_2_, strongly impaired *D*_LCO_ and progressive right heart failure [9]. CPFE displays a pattern of variable degrees of emphysema and fibrosis on CT. These patients seem to share clinical characteristics with IPAH with a lung phenotype [10].

Previous studies showed that even in patients with no or minimal changes in CT, histopathology may detect emphysematous and fibrotic changes [11]. However, as autopsies are rarely performed, verifying the existence of a vanishing pulmonary capillary syndrome histologically proved difficult.

Hyperpolarised gas MRI may assess lung microstructure and, e.g. emphysematous changes through increases in apparent diffusion coefficient (ADC) [12] noninvasively. Since xenon is taken up in lung tissue and blood and ^129^Xe experiences strong chemical shifts, ^129^Xe MRI is sensitive to alveolar-capillary diffusion and microvascular pulmonary blood volume [13]. With the appropriate choice of sequence parameters, ^129^Xe MRI metrics are specific to the gas uptake region and pulmonary capillaries as opposed to, e.g. dynamic contrast-enhanced MRI. Imaging of the ^129^Xe dissolved phase and dynamic acquisition of cardiogenic oscillations of ^129^Xe in red blood cells (RBC) may differentiate cardiopulmonary diseases and may provide information on pre- or postcapillary involvement in PH [14]. Although widespread clinical application is hampered by limited dissemination of necessary equipment, hyperpolarised ^129^Xe MRI is a useful tool for clinical research and the field of ^129^Xe MRI continues to grow.

We hypothesise that patients with IPAH and low *D*_LCO_ differ from other patient groups by a loss of pulmonary capillaries, as judged by the ratio of ^129^Xe in RBCs and membrane tissues (M). The purpose of this study was to test this hypothesis and to evaluate alveolar microstructure by comparing results from ^129^Xe MRI in IPAH with low *D*_LCO_ with those from IPAH with preserved *D*_LCO_, as well as with those from PH patients with CPFE and with those from patients with emphysema without echocardiographic signs of PH.

## Materials and methods

Ethical approval for this observational, prospective study was granted by the institutional review board of Hannover Medical School. MRI was performed at Hannover Medical School between 03/2019 and 06/2023. Participants gave written informed consent.

### Participants

Four patient groups were consecutively recruited from the outpatient clinic of the Department of Respiratory Medicine and Infectious Diseases of Hannover Medical School:

1. Patients with IPAH and preserved *D*_LCO_ (≥ 45% of predicted, based on findings in [4]) and forced expiratory volume in 1 s (FEV_1_) of ≥ 60% predicted;
2. Patients with IPAH and low *D*_LCO_ (< 45% predicted) and FEV_1_ ≥ 60% predicted without clinically significant parenchymal lung disease on CT;
3. Patients with CPFE on chest CT and PH;
4. Patients with emphysema without PH diagnosis.

Lung function testing, blood gas analyses, right-heart catheterisation (groups 1–3), as well as echocardiography (group 4) were part of clinical assessments. Right heart catheterisation was done at the time of PH diagnosis (except for patients with emphysema in whom echocardiography did not show signs of PH). PH was defined as mean pulmonary arterial pressure > 20 mmHg [1]. Clinical chest CT was available within 12 months. Exclusion criteria were pregnancy and MRI contraindications.

### ^129^Xe application

Locally produced hyperpolarised ^129^Xe (isotopically enriched ^129^Xe, polarisation 20–30%, Model 9810, Polarean) was authorised as a medical device for research applications. Doses were topped up with nitrogen to 1 L and dispensed into Tedlar bags (Jensen Inert Products). Subjects were instructed to exhale forcefully and inhale the dose in one breath. For the first two doses, in participants with vital capacity > 3 L, a second Tedlar bag containing air was connected to achieve a volume of 1/3 vital capacity [15]. For the last dose, participants were coached to further inhale room air after ^129^Xe to reach full lung inflation which may be beneficial for the repeatability of chemical shift saturation recovery (CSSR) measurements [16]. MRI was performed in breathhold. Vital signs were monitored by a physician during ^129^Xe applications.

### Imaging

^129^Xe MRI was performed at 1.5 T/17.6 MHz (Avanto, Siemens) using a linearly-polarised birdcage coil with a 16-channel receive array (Rapid Biomedical) and self-developed pulse sequences. Common sequence parameters are summarised in Table 1.

**Table 1:** Sequence parameters

Sequence	TR/ms	TE/ms	Flip angle/°	Reconstructed FOV/mm	Reconstructed matrix
Ventilation imaging	3.2	1.6	~10	384 × 384 × 384	96 × 96 × 96
Dissolved-phase imaging	18	Echo train 1: 0.61/2.23/3.85 Echo train 2: 1.42/3.04	21 (dissolved)/0.4 (gas)	320 × 320 × 320	48 × 48 × 48
Fixed-TR dynamic spectroscopy	36	1.2	60	n/a	n/a
CSSR	Variable	0.7	~27 (excitation using maximum RF power)	n/a	n/a
Diffusion-weighted imaging	57	41.6	7.4	400 × 400 × 240	64 × 64 × 6

The sequence parameters for dissolved-phase imaging are comparable to those previously established for multi-echo techniques and to ^129^Xe clinical trial consortium recommendations for multi-site trials [15, 28]. Compared to the recommendations, ventilation imaging uses a radial trajectory facilitating acceleration. Diffusion-weighted imaging uses shorter diffusion times to facilitate the estimation of surface-volume ratio. The impact of frequency-selective excitation in fixed-TR dynamic spectroscopy on dissolved-phase ratios is assessed in the supplemental material

The first dose was used for ventilation imaging using a 3D radial balanced steady-state free precession sequence and transmitter calibration. A second dose (600–800 mL ^129^Xe, dose equivalent to ~130 mL) was used for fixed-TR dynamic dissolved-phase spectroscopy and dissolved-phase imaging. A third dose (500–600 mL ^129^Xe) was used for diffusion-weighted imaging and CSSR with a combined imaging time of 11 s [17].

Dissolved-phase imaging [17] was performed using five echoes in the dissolved phase. Ratio maps M-gas, RBC-gas, and RBC-M were formed and whole-lung averages were calculated within an automatically generated mask based on gas-phase SNR. Whole-lung RBC-M was defined as the primary dependent variable.

Fixed-TR dynamic spectroscopy was not spatially selective and employed excitation at 222.5 ppm using a 2.2 ms frequency-selective radiofrequency pulse systematically increasing RBC signals. Sequence parameters included 512 points, a bandwidth of 16.7 kHz, and 160 measurements. Two Lorentzians including a phase term were fitted to real parts of spectra after zeroth order phasing corresponding to M and RBC. The median RBC-M chemical shift difference was determined. The function

1 $$f\left(n\right)=m\left(1+a\,{\mathrm{sin}}\left(r\,n+\varphi \right)\right)+d\left(n-\frac{{N}_{{{\rm{meas}}}}}{2}\right)$$

was fitted to the ratio of magnitudes to quantify the average RBC-M ratio (*m*) and oscillation amplitude (*a*). *N*_meas_ denotes the number of measurements (discarding the first 10%), *n* is the excitation number, *r* is proportional to heart rate, *φ* a phase factor and *d* accounts for signal drifts.

In CSSR spectroscopy, two 2.4 ms rectangular radiofrequency pulses saturated the dissolved-phase magnetisation. Dissolved-phase signal-buildup/gas uptake at delay times of 3–600 ms was probed using a 1.2 ms Gaussian pulse without localisation and free-induction decays sampled with bandwidth 16.7 kHz. ^129^Xe in the three compartments was quantified after zeroth- and first-order phasing by numerical integration of real parts. CSSR data were analysed by fitting a generalised model of ^129^Xe septal uptake [18] yielding membrane permeability κ defined as the ratio of diffusion coefficient and membrane thickness, describing delayed uptake to RBCs, RBC fraction η derived from relative RBC signal intensity at the time of saturation of alveolar septa with ^129^Xe, as well as capillary transit time τ derived from RBC signal increase at high delay times.

From diffusion-weighted ^129^Xe MRI, whole-lung time-dependent ADCs were calculated and the whole-lung ratio of alveolar surface to gas volume quantified from data with 920 µs, 1140 µs, 1400 µs, 1660 µs, and 1960 µs diffusion time with 11, 9, 8, 7, and 7 repetitions of the diffusion-sensitising gradients at a constant *b*-value of 3 s/cm^2^. A second-order approximation of ADC time dependence was assumed [19].

Chest CT was evaluated by a radiologist (J.V.C., 21 years of experience). Lungs were segmented and low-attenuation area percentage (voxels with attenuation ≤ −950 HU) was quantified automatically using Aview (version 1.1.42.53, Coreline Soft).

### Statistical analysis

Statistical analysis was performed by AK using subroutines in R (version 4.1.2). Kruskal–Wallis tests were performed to assess differences between groups. Post-hoc analysis was performed using Dunn tests including Holm *p*-value adjustment (kwManyOneDunnTest, package PMCMRplus, version 1.9.10) to test pairwise differences between IPAH with low *D*_LCO_ and all other groups. Correlations were assessed using Pearson’s correlation. The significance level was 0.05 two-sided. Results are presented as median (interquartile range).

## Results

### Participants

Out of the 29 initially recruited participants 7 were excluded resulting in 22 participants for analysis: 5 participants with IPAH and preserved *D*_LCO_, 5 participants with IPAH and low *D*_LCO_, 7 PH participants with CPFE and 5 participants with pulmonary emphysema without PH. Results for exclusion were notable emphysema or parenchymal lung disease on chest CT in patients of the IPAH low *D*_LCO_ group (four cases) or suspected pulmonary veno-occlusive disease based on their clinical phenotype (two cases) or histological results (one case). Data from diffusion-weighted imaging and CSSR could not be evaluated in one CPFE patient due to inadequate SNR.

Table 2 summarises participant demographics and clinical characteristics. The median interval between right heart catheterisation and MRI was 1.1 years. Participants with IPAH and preserved *D*_LCO_ were comparatively young (median 55 years), the majority never smoked. FEV_1_ and *D*_LCO_ were preserved with near-normal PaO_2_ and low PaCO_2_. Right-heart catheterisation at PH diagnosis showed severe pre-capillary PH. Patients received double (four cases) or triple (one case) combination therapy for PH. Four patients received macitentan, three received a PDE5 inhibitor, two riociguat, one selexipag, and one imatinib.

**Table 2:** Participant demographics and clinical characteristics

	All patients	IPAH preserved *D*_LCO_	IPAH low *D*_LCO_ (lung phenotype)	CPFE	Emphysema
Number	22	5	5	7	5
Age at study, years	65 (57–71)	55 (43–59)	65 (61–72)	71 (63–79)	66 (62–70)
Sex, male, %	50	60	40	71	20
*D*_LCO,_ % pred.	33 (28–40)	72 (66–82)	29 (27–31)	33 (24–38)	29 (19–36)
FEV_1_, % pred.	73 (63–94)	94 (87–94)	74 (71–92)	81 (66–94)	39 (23–47)
FVC, % pred.	94 (79–104)	89 (88–110)	98 (93–102)	80 (79–103)	71 (70–102)
TLC, % pred.	100 (84–107)	89 (83–102)	100 (93–105)	86 (67–103)	113 (106–127)
RV, % pred.	106 (85–137)	86 (85–98)	121 (88–131)	96 (61–132)	173 (156–213)
Body mass index, kg/m^2^	26 (24–29)	25 (21–28)	27 (26–34)	27 (25–29)	22 (21–26)
World Health Organization functional class					
I, *n* (%)	2 (12)	2 (40)	0 (0)	0 (0)	/
II, *n* (%)	4 (24)	2 (40)	1 (20)	1 (14)	/
III, *n* (%)	10 (59)	1 (20)	3 (60)	6 (86)	/
IV, *n* (%)	1 (6)	0 (0)	1 (20)	0 (0)	/
6-min walk distance, m	346 (251–387)	558 (484–546)	252 (177–335)	353 (250–373)	/
N-terminal pro-B-type natriuretic peptide, ng/L	279 (69–1576)	60 (40–141)	306 (132–2675)	1212 (643–2786)	/
Haemoglobin, g/dL	15.5 (14.3–16.9)	15.4 (14.4–17.0)	15.8 (13.6–16.7)	15.2 (14.5–16.5)	15.6 (14.9–17.4)
Arterial partial pressure of oxygen, mmHg	61 (54–72)	77 (65–79)	57 (54–62)	58 (53–62)	60 (55–68)
Partial pressure of carbon dioxide, mmHg	36 (34–39)	34 (33–39)	35 (32–40)	34 (33–36)	39 (35–42)
Oxygen saturation, %	92 (90–95)	96 (95–97)	91 (88–93)	91 (88–92)	92 (92–95)
Oxygen supplementation, *n* (%)	13 (59)	0 (0)	4 (80)	5 (71)	4 (80)
Smoking status, *n* (%)					
Active	3 (14)	0 (0)	1 (20)	2 (29)	0 (0)
Former	15 (68)	2 (40)	3 (60)	5 (71)	5 (100)
Never	4 (18)	3 (60)	1 (20)	0 (0)	0 (0)
Pack years	42 (5–50)	0 (0–3)	30 (23–44)	50 (45–65)	40 (13–49)
Haemodynamics					
Right atrial pressure, mmHg	10 (7–12)	8 (5–9)	10 (7–12)	12 (8–13)	/
Mean pulmonary arterial pressure, mmHg	47 (40–54)	53 (42–59)	44 (43–48)	47 (36–54)	/
Pulmonary arterial wedge pressure, mmHg	11 (8–14)	9 (8–11)	14 (10–15)	11 (6–15)	/
Cardiac index, L/min/m^2^	2.2 (2.0–2.4)	1.8 (1.6–1.9)	2.3 (2.2–2.6)	2.2 (2.0–2.4)	/
Pulmonary vascular resistance, Wood units	8 (6–13)	14 (8–16)	7 (7–8)	9 (5–11)	/
Mixed venous oxygen saturation, %	63 (57–67)	67 (56–68)	60 (58–66)	54 (42–66)	/

Values describing a distribution are shown as median (interquartile range)

*CPFE* combined pulmonary fibrosis and emphysema, *D*_*LCO*_ diffusion capacity of the lung for carbon monoxide, *FEV*_*1*_ forced expiratory volume in 1 s, *FVC* forced vital capacity, *IPAH* idiopathic pulmonary arterial hypertension, *RV* residual volume, *TLC* total lung capacity

Patients with IPAH and low *D*_LCO_ were comparatively older (median 65 years) and had a substantial smoking history (median 30 pack-years). FEV_1_ was mildly reduced, *D*_LCO_ was severely reduced, and these participants were more hypoxaemic. These participants also had severe PH comparable to participants with IPAH and preserved *D*_LCO_. All patients were treated with a PDE5 inhibitor, two also received macitentan.

Patients with CPFE and PH were similar in age to patients with IPAH and low *D*_LCO_ and predominantly male. Patients with CPFE and PH also had a substantial smoking history (median 50 pack-years). *D*_LCO_ was similar to patients with IPAH and low *D*_LCO_. All patients were treated with a PDE5 inhibitor, one additionally with macitentan and selexipag.

Patients with emphysema without PH diagnosis, similar in age to patients with IPAH and low *D*_LCO_, had markedly reduced FEV_1_ and presented with comparable, very low *D*_LCO_ (median 29% pred.). Echocardiography showed no signs of PH in these patients.

### Imaging

**Fig. 1:**
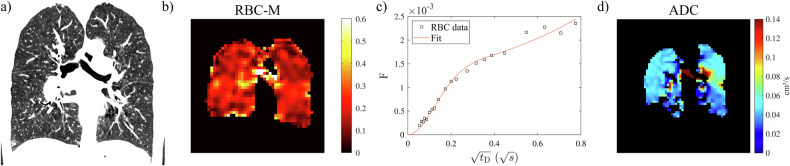
Representative participant with IPAH and preserved *D*_LCO_ (67-year-old female, *D*_LCO_ 82% pred., no smoking history): (**a**) CT (low-attenuation area percentage 3.0%), (**b**) ratio of ^129^Xe in RBC and M (whole-lung value 0.144) from ^129^Xe dissolved-phase MRI, (**c**) uptake of ^129^Xe to RBCs normalised by a gas signal as a function of delay time *t*_D_ in CSSR (η = 0.28, τ = 0.91 s), and (**d**) ADC at 3 ms diffusion time from diffusion-weighted imaging. The surface/volume ratio from diffusion-weighted ^129^Xe MRI for this participant is 221 cm^−1^. ADC, apparent diffusion coefficient; CSSR, chemical shift saturation recovery; *D*_LCO_, diffusion capacity of the lung for carbon monoxide; IPAH, idiopathic pulmonary arterial hypertension; M, membrane tissues; RBC, red blood cell; *t*_D_, delay time

**Fig. 2:**
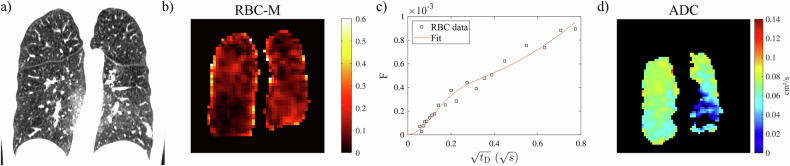
Representative participant within the group IPAH low *D*_LCO_ (64-year-old female, *D*_LCO_ 32% pred., 30 pack-years): (**a**) CT (low-attenuation area percentage 1.3%), (**b**) RBC-M ratio maps from ^129^Xe dissolved-phase MRI (whole-lung value 0.054), (**c**) CSSR uptake to RBC (RBC fraction η = 0.10, capillary transit time τ = 0.44 s), and (**d**) ADC at 3 ms diffusion time. RBC-M is diffusely low corresponding to low RBC uptake in CSSR at intermediate delay whereas ADC is mostly increased throughout the lung parenchyma. The surface/volume ratio from diffusion-weighted ^129^Xe MRI for this participant is 151 cm^–1^. ADC, apparent diffusion coefficient; CSSR, chemical shift saturation recovery; *D*_LCO_, diffusion capacity of the lung for carbon monoxide; IPAH, idiopathic pulmonary arterial hypertension; M, membrane tissues; RBC, red blood cell; *t*_D_, delay time

**Fig. 3:**
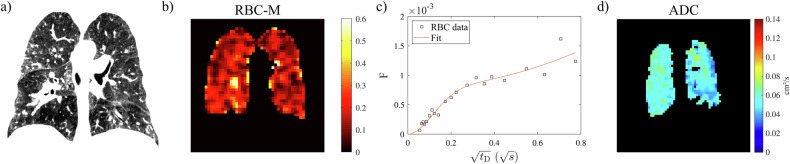
Representative participant with CPFE (61-year-old male, *D*_LCO_ 39% pred., 70 pack-years): (**a**) CT (low-attenuation area percentage 10.9%), (**b**) ratio of ^129^Xe in RBC and M (whole-lung value 0.117) from ^129^Xe dissolved-phase MRI, (**c**) uptake of ^129^Xe to RBCs normalised by a gas signal as a function of delay time *t*_D_ in CSSR (η = 0.16, τ = 0.82 s) and (**d**) ADC at 3 ms diffusion time from diffusion-weighted imaging. The surface/volume ratio from diffusion-weighted ^129^Xe MRI for this participant is 216 cm^–1^. ADC, apparent diffusion coefficient; CPFE, combined pulmonary fibrosis and emphysema; CSSR, chemical shift saturation recovery; *D*_LCO_, diffusion capacity of the lung for carbon monoxide; M, membrane tissues; RBC, red blood cell, *t*_D_, delay time

**Fig. 4:**
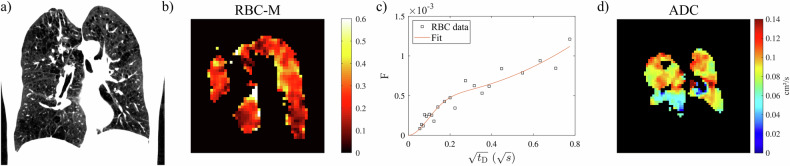
Representative participant with emphysema (63-year-old female, *D*_LCO_ % pred., 16 pack-years): (**a**) CT (low-attenuation area percentage 24.2%), (**b**) ratio of ^129^Xe in RBC and M (whole-lung value 0.190) from ^129^Xe dissolved-phase MRI, (**c**) uptake of ^129^Xe to RBCs normalised by a gas signal as a function of delay time *t*_D_ in CSSR (η = 0.22, τ = 0.49 s), and (**d**) ADC at 3 ms diffusion time from diffusion-weighted imaging. The surface/volume ratio from diffusion-weighted ^129^Xe MRI for this participant is 138 cm^–1^. ADC, apparent diffusion coefficient; CSSR, chemical shift saturation recovery; *D*_LCO_, diffusion capacity of the lung for carbon monoxide; M, membrane tissues; RBC, red blood cell; *t*_D_, delay time

**Fig. 5:**
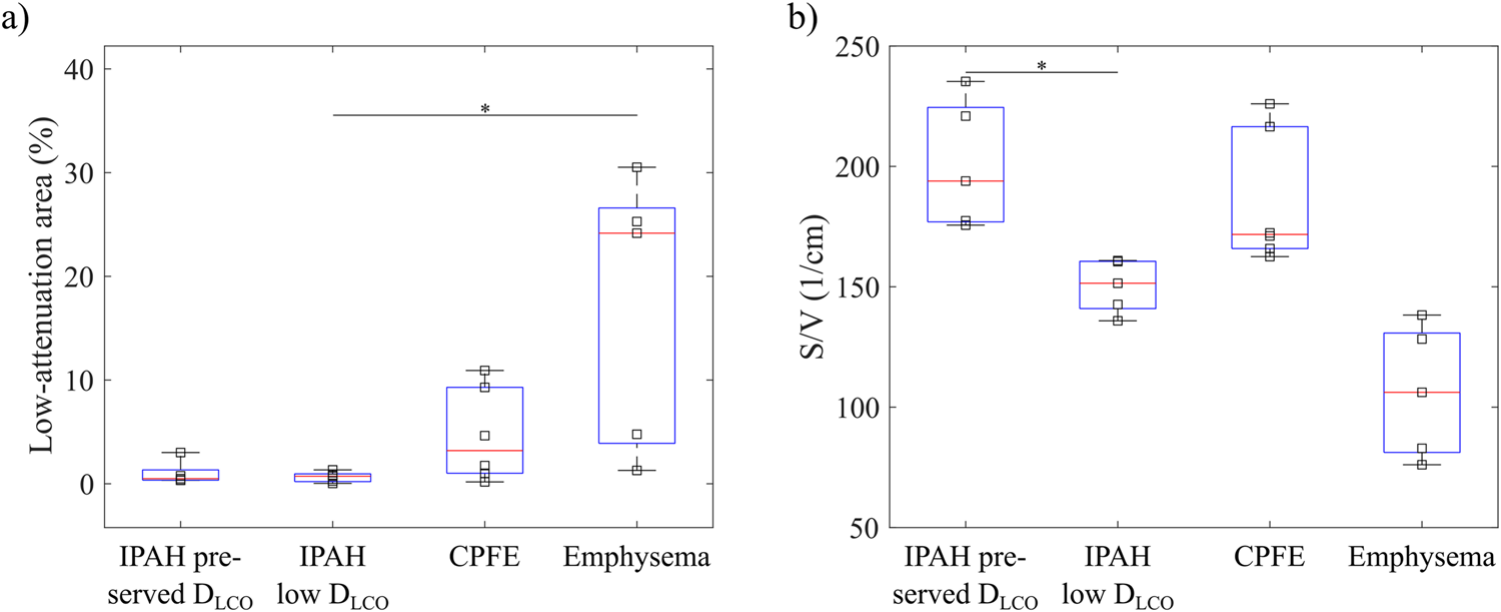
**a** Low attenuation area percentage in the different study groups. **b** Lung S/V ratio from diffusion-weighted imaging in all four study groups. Significant differences between the group IPAH and low *D*_LCO_ with all other groups from post-hoc analysis are marked by asterisks. CPFE, combined pulmonary fibrosis and emphysema; *D*_LCO_, diffusion capacity of the lung for carbon monoxide; IPAH, idiopathic pulmonary arterial hypertension; S/V, surface/volume

Representative CT images for participants of all groups are depicted in Figs. 1a–4a, respectively. Low-attenuation area percentages are shown in Fig. 5a. Values were significantly different between groups (*p* = 0.02) but not increased in the IPAH with low *D*_LCO_ group compared to other groups.

Lung surface-volume ratio from time-dependent diffusion measurements was significantly different between groups (*p* < 0.001), see Fig. 5b. In contrast to low-attenuation area percentage, the surface-volume ratio was significantly reduced in participants of the IPAH with low *D*_LCO_ group compared to the IPAH with preserved *D*_LCO_ group (*p* = 0.04). The median surface-volume ratio was lowest in the emphysema group.

**Fig. 6:**
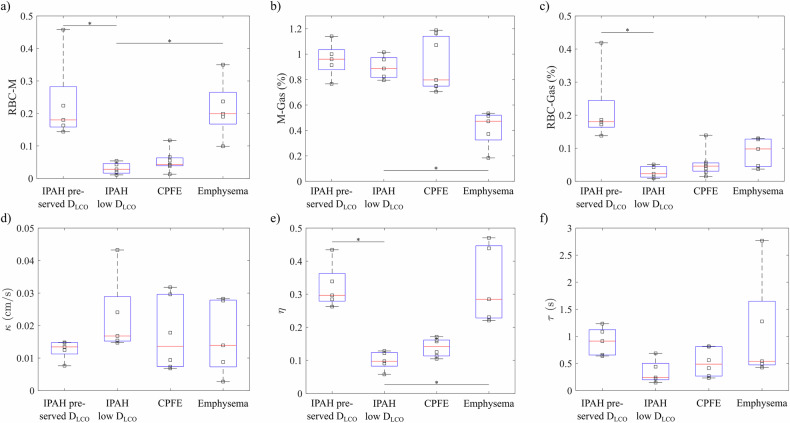
**a** RBC-M, (**b**) M-gas, and (**c**) RBC-gas ratio from dissolved-phase imaging, as well as (**d**) membrane permeability κ, (**e**) RBC fraction η, as well as (**f**) capillary transit time τ from CSSR spectroscopy. Significant differences between the group IPAH and low *D*_LCO_ with all other groups from post-hoc analysis are marked by asterisks. CPFE, combined pulmonary fibrosis and emphysema; *D*_LCO_, diffusion capacity of the lung for carbon monoxide; IPAH, idiopathic pulmonary arterial hypertension; M, membrane tissues; RBC, red blood cell

Representative ratio maps of ^129^Xe in RBC and M from dissolved-phase imaging are shown in Figs. 1b–4b. The RBC-M ratio was diffusely low throughout the lung parenchyma in the IPAH with low *D*_LCO_ group (Fig. 2b). Whole-lung averages were significantly different between groups (*p* = 0.001), Fig. 6a. In post-hoc analysis, RBC-M was significantly reduced in the IPAH with low *D*_LCO_ group compared to both the IPAH with preserved *D*_LCO_ group (*p* = 0.006) and the emphysema group (*p* = 0.006). No significant difference was observed in comparison to the CPFE group (*p* = 0.45).

The RBC fraction η from CSSR was significantly different between the groups (*p* = 0.001), Fig. 6e. η was significantly reduced in the IPAH with low *D*_LCO_ group compared to the IPAH with preserved *D*_LCO_ group (*p* = 0.004) and to the emphysema group (*p* = 0.004). No difference was observed with respect to the CPFE group (*p* = 0.33). The parameters κ and τ were not significantly different between the four groups (*p* = 0.36 and 0.05). Interestingly, capillary transit time τ tended to be lower in the IPAH with low *D*_LCO_ (0.24 s (0.20–0.50 s)) than in the IPAH with preserved *D*_LCO_ group (0.91 s (0.66–1.12 s)), Fig. 6f.

Results from fixed-TR dynamic spectroscopy of the ^129^Xe dissolved phase are summarised in supporting Fig. 1. Average RBC-M was different between groups (*p* = 0.002) and reduced in IPAH with low *D*_LCO_ compared to IPAH with preserved *D*_LCO_ (*p* = 0.02) and the emphysema group (*p* = 0.03). Oscillation amplitudes were significantly different between groups (*p* = 0.02) but post-hoc analysis showed no significant differences. Both IPAH groups tended to have low oscillation amplitudes. The RBC-M chemical shift difference was significantly different between groups (*p* = 0.002) and reduced in the IPAH with low *D*_LCO_ group compared to the IPAH with preserved *D*_LCO_ group (*p* = 0.005).

Ventilation defect percentage was significantly different between groups (*p* = 0.02) and highest in the emphysema group, but not different between IPAH with low *D*_LCO_ compared to IPAH with preserved *D*_LCO_ (*p* = 0.67).

### Correlations

Cardiac index from right-heart catheterisation and capillary transit time τ were inversely correlated in the first three groups (*R* = −0.64, *p* = 0.008). There was a moderate correlation between CT-derived low-attenuation area and surface-volume ratio from ^129^Xe MRI (*R* = −0.45, *p* = 0.04). RBC-M chemical shift difference was correlated with oxygen partial pressure from blood gas analysis (*R* = 0.55, *p* = 0.006). RBC-M from fixed-TR dynamic spectroscopy was strongly correlated with whole-lung averages of imaging (*R* = 0.98, *p* < 0.001), supporting Fig. 2, and with η (*R* = 0.81, *p* < 0.001), but not with κ (*R* = −0.17, *p* = 0.45). Surface-volume ratio from diffusion-weighted imaging was strongly correlated with whole-lung M-Gas from dissolved-phase imaging (*R* = 0.82, *p* < 0.001) but not RBC-M (*R* = −0.18, *p* = 0.44).

## Discussion

This study was performed to non-invasively detect a potential loss of pulmonary capillaries in patients with IPAH and low *D*_LCO_, as well as to evaluate lung microstructure. We found a significantly reduced RBC-M ratio, as well as RBC fraction η in the IPAH with a low *D*_LCO_ group compared to both the preserved *D*_LCO_ group and emphysema groups. At the same time, the lung surface-volume ratio derived from ^129^Xe ADCs in lung airspaces was significantly decreased in IPAH with low *D*_LCO_ compared to IPAH with preserved *D*_LCO_ although CT did not show signs of clinically significant macroscopic emphysema in IPAH with low *D*_LCO_.

These findings support the hypothesis of a loss of pulmonary capillaries in participants of the IPAH with low *D*_LCO_ group, as seen by the significant reduction in RBC-M and the RBC fraction η. Furthermore, results from ^129^Xe MRI also suggest early emphysematous changes in IPAH with low *D*_LCO_ as seen by the reduced lung surface-volume ratio despite negligible low-attenuation area on CT. This indicates that alveolar tissue destruction as part of early diffuse emphysema is likely contributing to *D*_LCO_ reduction in IPAH with low *D*_LCO_. Four of five participants with IPAH and low *D*_LCO_ were former or active smokers. One participant never smoked but was a metalworker with occupational exposure to toxic fumes for several decades.

Our findings suggest that the IPAH with low *D*_LCO_ group mainly reflects the vascular phenotype of COPD, corresponding to World Health Organization PH group 3, with predominantly pulmonary capillary loss or dysfunction with relatively mild FEV_1_ reduction and diffuse alveolar tissue destruction as part of early emphysema leading to severe alveolar membrane dysfunction with severely reduced *D*_LCO_ and resulting hypoxia. Measured pathophysiological changes are summarised in Table 3 with reference values from healthy subjects [17].

**Table 3:** Summary of pathophysiological changes

Quantity	IPAH preserved *D*_LCO_	IPAH low *D*_LCO_ (lung phenotype)	CPFE	Emphysema
Airspace enlargement				
LAA (CT)	0	0	+	++
S/V (^129^Xe MRI)	0	−	(−)	−−
Capillary rarefication				
RBC fraction η	0	−	−	0
RBC-TP	0	−	−	0
Capillary blood flow				
Capillary transit time τ	+	−	0	0
RBC oscillation amplitude	−	−	0	0
Alveolar membrane function				
Membrane permeability κ	−	−	−	−
RBC chemical shift (oxygenation)	0	−−	−−	−
Ventilation heterogeneity				
Ventilation defect percentage	+	+	+	++
FEV_1_ as % of predicted value	0	−	−	−−

Reference values for parameters from CSSR measurements in a group of 12 healthy volunteers reported in [17] (7 male/5 female, age 49 years ± 14 years) are κ: 0.051 cm/s ± 0.023 cm/s, η: 0.247 ± 0.041, τ: 0.67 s ± 0.18 s. Similarly, for fixed-TR dynamic spectroscopy, reference values are average RBC-M: 1.04 ± 0.17, RBC oscillation amplitude: 3.5% ± 0.5%, RBC chemical shift 18.7 ppm ± 0.4 ppm

++ strongly increased, + increased, 0 equal, − reduced, −− strongly reduced, *CPFE* combined pulmonary fibrosis and emphysema, *D*_*LCO*_ diffusion capacity of the lung for carbon monoxide, *FEV*_*1*_ forced expiratory volume in 1 s, *GP* gas phase, *IPAH* idiopathic pulmonary arterial hypertension, *LAA* low-attenuation area, *M* membrane tissues, *RBC* red blood cell, *S/V* surface/volume

The different findings from CT and MRI may suggest an increased sensitivity of ^129^Xe diffusion-weighted MRI for early emphysema detection. Recent work showed the potential of ^129^Xe diffusion-weighted imaging to detect airspace enlargement in normal-appearing areas of the fibrotic lung [20]. ^129^Xe diffusion-weighted imaging and assessment of RBC signal may add value to patient phenotyping.

Results from fixed-TR dynamic spectroscopy of the dissolved phase are consistent with those from dissolved-phase imaging despite the systematic difference in RBC-M owing to frequency-selective excitation and an offset in the scatter plot (supporting Fig. 2), possibly due to T_2_* differences or non-Lorentzian line-shapes. The fact that RBC-M strongly correlates with η but not κ suggests that RBC-M in these patients is relatively unaffected by membrane permeability and mostly reflective of capillary density within alveolar tissue. The relative RBC signal from dissolved-phase imaging has been suggested to be reflective of capillary volume in analogy to the Roughton–Forster model [21].

Post-hoc analysis of RBC oscillation amplitudes showed no significant differences likely due to limited sample size. There was a trend towards reduced oscillation amplitudes in precapillary PAH compared to patients with emphysema without PH. The reduced RBC-M chemical shift difference in patients with IPAH and low *D*_LCO_ is likely due to reduced oxygenation [22]. Furthermore, we observed a tendency for higher capillary flow in the IPAH with low *D*_LCO_ group compared to the IPAH group with preserved *D*_LCO_. This is consistent with the hypothesis of capillary loss leading to increased capillary flow in remaining capillaries, shortening the oxygenation time. However, the influence of cardiac index on capillary transit time should be considered given that data from right-heart catheterisation showed a higher cardiac index in the IPAH with a low *D*_LCO_ group on average. Interestingly, both capillary transit time and the product of RBC fraction η and surface-volume ratio were lower in IPAH with low *D*_LCO_ compared to emphysema patients without PH, possibly reflecting a larger loss of capillary surface area.

### Limitations

Measures of ^129^Xe gas uptake, particularly RBC-M, depending on age and sex in healthy subjects with increased values in males and younger age [23]. In addition, dissolved-phase ratios depend on haemoglobin [24] and could potentially also depend on oxygenation [25]. Future work should concentrate on the further establishment of reference values, as well as on the exact influence of confounding variables. Correlations between invasive haemodynamics and MRI findings must be interpreted with caution as these measurements were not obtained simultaneously and PAH treatments were initiated in between, potentially affecting pulmonary blood flow.

The parameters from the generalised CSSR model in [18] have not been validated by comparison to gold standard measurements. This study showed a correlation of capillary transit time with cardiac index. Results for κ may be affected by noise and uncertainties in transmitter calibration.

The inability of some participants to hold their breath long enough may have influenced the results. In particular, imprecision and bias may arise from the dependence of dissolved-phase ratios on lung inflation [26]. This was partly addressed by administering gas volumes normalised to spirometry. Previous studies suggest an influence of lung size which possibly cannot be addressed by standardised breathing manoeuvres [27].

The generalizability of the results of this single-centre study to other centres with different patient cohorts and imaging methods is an open question. Multi-centre studies with larger sample sizes are needed but are hampered by limited dissemination of ^129^Xe MRI.

## Conclusion

Our results are consistent with a loss of pulmonary capillaries in patients with IPAH and low *D*_LCO_ in conjunction with the destruction of alveolar membranes, likely due to early diffuse emphysema.

## Supplementary information

ELECTRONIC SUPPLEMENTARY MATERIAL

## Abbreviations

ADC: Apparent diffusion coefficient
COPD: Chronic obstructive pulmonary disease
CPFE: Combined pulmonary fibrosis and emphysema
CSSR: Chemical shift saturation recovery
*D*_LCO_: Diffusion capacity of the lung for carbon monoxide
FEV_1_: Forced expiratory volume in 1 s
IPAH: Idiopathic pulmonary arterial hypertension
M: Membrane tissues
PH: Pulmonary hypertension
RBC: Red blood cell
