# Supplementary material for: Loss of pulmonary capillaries in idiopathic pulmonary arterial hypertension with low diffusion capacity is accompanied by early diffuse emphysema detected by 129Xe MRI
Source: Eur Radiol. 2024 Dec 8;35(6):3010–20. doi: 10.1007/s00330-024-11209-1 (PMC12081569; doi:10.1007/s00330-024-11209-1)
Supplement: Supplementary file 1 — ELECTRONIC SUPPLEMENTARY MATERIAL [file 330_2024_11209_MOESM1_ESM.docx]

**Loss of pulmonary capillaries in idiopathic pulmonary arterial hypertension with low diffusion capacity is accompanied by early diffuse emphysema detected by ^129^Xe MRI**

**– Supporting Material –**


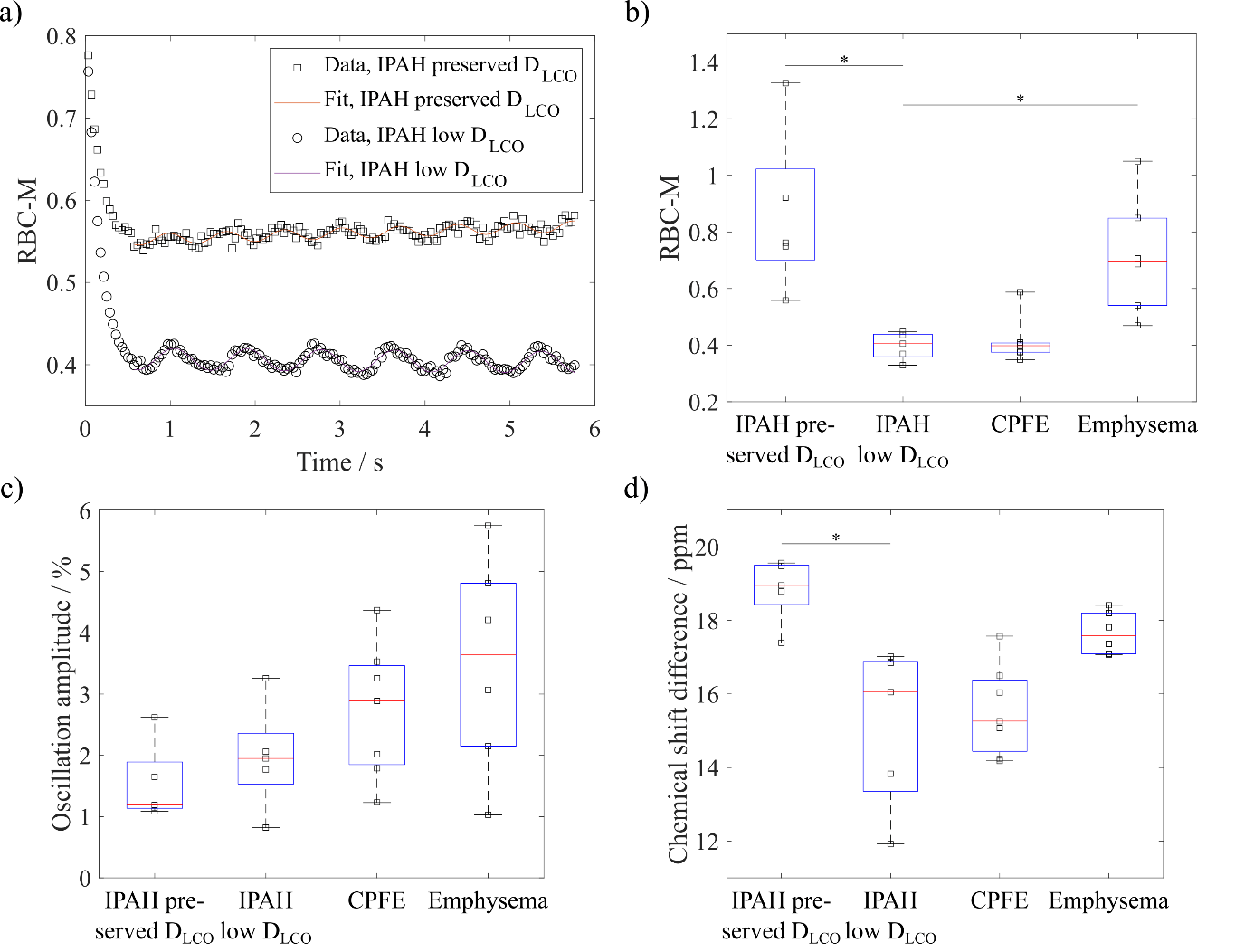


Supporting figure 1. a) Representative data for RBC-M from fixed-TR dynamic spectroscopy in the participants with IPAH and preserved (squares)/low (circles) D_LCO_ presented in figures 1 and 2. Cardiogenic oscillations have larger amplitude in the participant with IPAH and low D_LCO_. Boxplots for data of all subjects for b) average RBC-M ratio, c) amplitude of RBC oscillations and d) chemical shift difference between RBC and M as median over all measurements. RBC-M ratios show essentially the same differences between groups as in dissolved-phase imaging. Oscillation amplitude tends to be increased in emphysema patients. RBC-M chemical shift difference is significantly reduced in participants with IPAH and low D_LCO_. Significant differences between the group IPAH and low D_LCO_ with all other groups from post-hoc analysis are marked by asterisks. Abbreviations: CPFE – combined pulmonary fibrosis and emphysema, D_LCO_ – diffusion capacity of the lung for carbon monoxide, IPAH – idiopathic pulmonary arterial hypertension, M – membrane tissues, RBC – red blood cell.

**
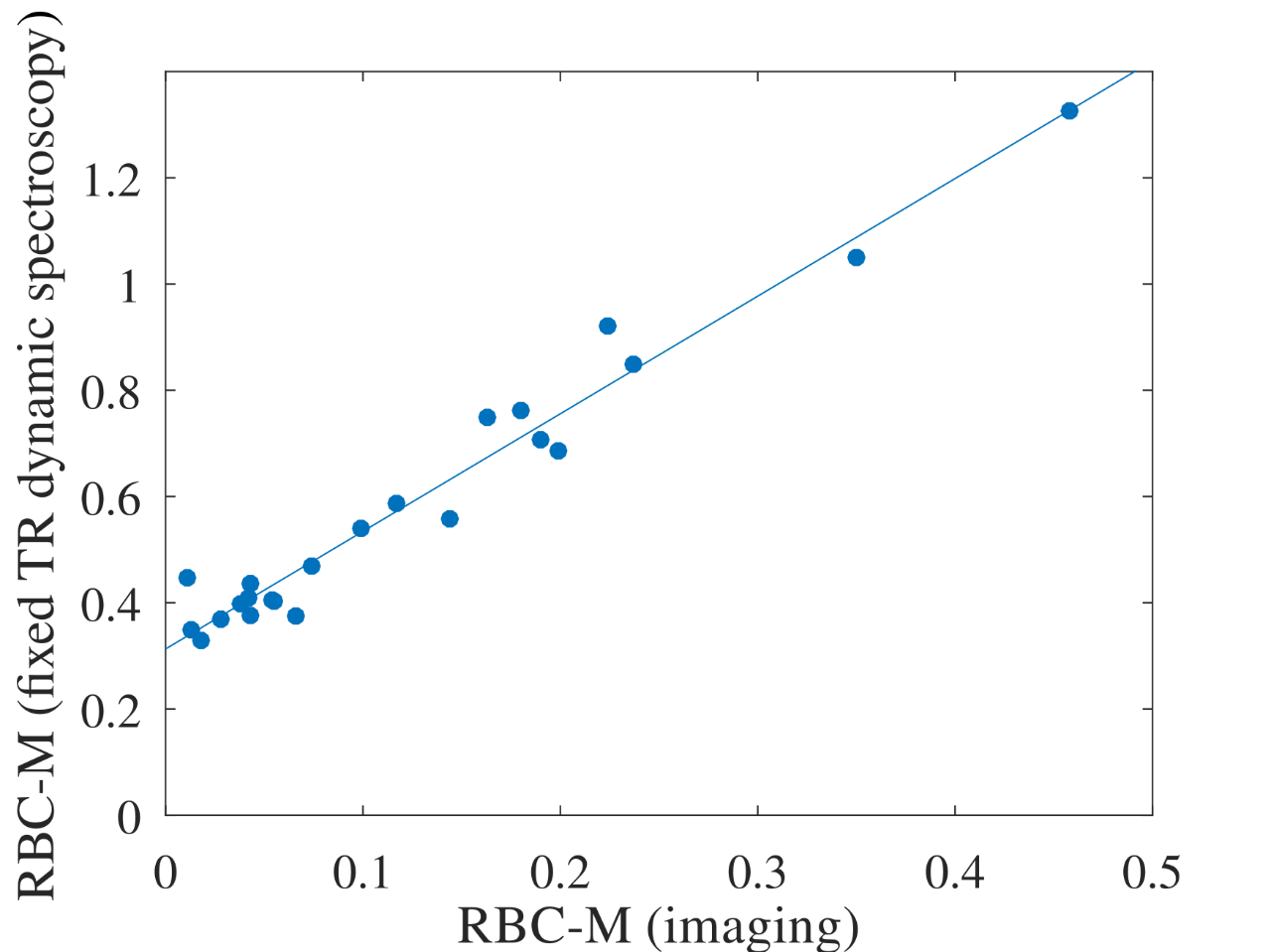
**

Supporting figure 2. Correlation of RBC-M from dissolved-phase imaging with average RBC-M from fixed-TR dynamic spectroscopy employing frequency-selective excitation to enhance relative RBC signal. A strong correlation is observed (R = 0.98, p < 0.001). Also shown is a linear fit with slope 2.214 and vertical intercept 0.313. Abbreviations: M – membrane tissues, RBC – red blood cell, TR – repetition time.
